# Cryptic extinction risk in a western Pacific lizard radiation

**DOI:** 10.1007/s10531-022-02412-x

**Published:** 2022-05-25

**Authors:** Peter J. McDonald, Rafe M. Brown, Fred Kraus, Philip Bowles, Umilaela Arifin, Samuel J. Eliades, Robert N. Fisher, Maren Gaulke, L. Lee Grismer, Ivan Ineich, Benjamin R. Karin, Camila G. Meneses, Stephen J. Richards, Marites B. Sanguila, Cameron D. Siler, Paul M. Oliver

**Affiliations:** 1 Secretariat of the Pacific Regional Environment Programme, PO Box 240, Apia, Samoa; 2 Flora and Fauna Division, Department of Environment, Parks, and Water Security, Northern Territory Government, Alice Springs, NT 0870 Australia; 3 Department of Ecology and Evolutionary Biology & Biodiversity Institute, University of Kansas, 1345 Jayhawk Boulevard, Lawrence, KS 66044 USA; 4 Department of Ecology and Evolutionary Biology, University of Michigan, Ann Arbor, MI USA; 5 Biodiversity Assessment Unit, International Union for Conservation of Nature and Conservation International, Washington, DC 20009 USA; 6 Universität Hamburg, Edmund-Siemers-Allee 1, 20148 Hamburg, Germany; 7 Leibniz Institute for the Analyses of Biodiversity Change, Zoological Museum Hamburg, Martin-Luther-King-Platz 3, 20146 Hamburg, Germany; 8 Museum of Vertebrate Zoology and Department of Integrative Biology, University of California, Berkeley, CA 94720 USA; 9 Sam Noble Oklahoma Museum of Natural History and Department of Biology, University of Oklahoma, Norman, OK 73072 USA; 10 U.S. Geological Survey, Western Ecological Research Center, 4165 Spruance Road, Suite 200, San Diego, CA 92101 USA; 11 Institut de Systématique, Évolution, Biodiversité (ISYEB) - Muséum National d’Histoire Naturelle, Sorbonne Université, École Pratique des Hautes Études, Université des Antilles, CNRS - CP 30, 57 rue Cuvier, 75005 Paris, France; 12 GeoBio-Center, Ludwig-Maximilians-University, Richard-Wagner-Str. 10, 80333 Munich, Germany; 13 Department of Biology, La Sierra University, 4500 Riverwalk Parkway, Riverside, CA 92505 USA; 14 Biodiversity Institute and Department of Ecology and Evolutionary Biology, University of Kansas, Lawrence, KS 66045 USA; 15 Department of Herpetology, South Australian Museum, North Terrace, Adelaide, SA 5000 Australia; 16 Biodiversity Informatics and Research Center and Natural Sciences and Mathematics Division, Arts and Sciences Program, Father Saturnino Urios University, Agusan del Norte, 8600 Butuan City, Philippines; 17 Centre for Planetary Health and Food Security, Griffith University, 170 Kessels Rd, Nathan, QLD 4111 Australia; 18 Biodiversity and Geosciences Program, Queensland Museum, South Brisbane, QLD 4101 Australia

**Keywords:** Geckos, IUCN red list, *Lepidodactylus*, Linnean shortfall, New Guinea, Philippines, Wallacea

## Abstract

Cryptic ecologies, the Wallacean Shortfall of undocumented species’ geographical ranges and the Linnaean Shortfall of undescribed diversity, are all major barriers to conservation assessment. When these factors overlap with drivers of extinction risk, such as insular distributions, the number of threatened species in a region or clade may be underestimated, a situation we term ‘cryptic extinction risk’. The genus *Lepidodactylus* is a diverse radiation of insular and arboreal geckos that occurs across the western Pacific. Previous work on *Lepidodactylus* showed evidence of evolutionary displacement around continental fringes, suggesting an inherent vulnerability to extinction from factors such as competition and predation. We sought to (1) comprehensively review status and threats, (2) estimate the number of undescribed species, and (3) estimate extinction risk in data deficient and candidate species, in *Lepidodactylus*. From our updated IUCN Red List assessment, 60% of the 58 recognized species are threatened (n = 15) or Data Deficient (n = 21), which is higher than reported for most other lizard groups. Species from the smaller and isolated Pacific islands are of greatest conservation concern, with most either threatened or Data Deficient, and all particularly vulnerable to invasive species. We estimated 32 undescribed candidate species and linear modelling predicted that an additional 18 species, among these and the data deficient species, are threatened with extinction. Focusing efforts to resolve the taxonomy and conservation status of key taxa, especially on small islands in the Pacific, is a high priority for conserving this remarkably diverse, yet poorly understood, lizard fauna. Our data highlight how cryptic ecologies and cryptic diversity combine and lead to significant underestimation of extinction risk.

**Supplementary Information:**

The online version contains supplementary material available at 10.1007/s10531-022-02412-x.

## Introduction

Scientists and conservationists have invested substantial effort assessing the status of significant portions of the world’s biodiversity (Rodrigues et al. 2006). Species-level conservation assessment is a first principle of all conservation planning, prioritization, and investment. For vertebrate taxa (which are generally better known) three key major impediments to determining conservation status and identifying declines have emerged. First, for rare species or those with cryptic ecologies (i.e. difficult-to-access microhabitats), sampling is frequently difficult, resulting in a lack of data necessary for conservation assessments, even under basic criteria such as extent of occurrence (McDonald 2004; Gillespie et al. 2020). For these difficult-to-sample species, population monitoring can be uninformative or prohibitively expensive, leaving conservation practitioners unable to measure population trends or the effectiveness of their management activities (Chadès et al. 2008). Second, the Wallacean Shortfall refers to the lack information on species’ distributions (Lomolino 2004), which impedes area-based assessment of conservation status. Third, is the Linnaean Shortfall (Brown and Lomolino 1998) or taxonomic impediment; that is, a large proportion of species diversity remains scientifically undocumented, with estimates ranging from 5 to 80 million unnamed species (NSB 1989). Listing of undescribed species on the International Union for Conservation of Nature (IUCN) Red List is discouraged (IUCN 2021) and knowledge of the distribution (i.e. the Wallacean Shortfall) and habitat requirements of candidate species (undescribed but recognized taxa) is frequently especially scant. Where threatened clades contain many difficult-to-sample, poorly known and/or undescribed species, there is a higher probablity that extinction risk will be underestimated, a situation that we term cryptic extinction risk (CER).

Although CER is, by its nature, hard to document, focused analyses of clades or regions where Linnaean Shortfall and/or cryptic ecologies are likely to operate provide opportunities to predict and highlight species groups and areas wherein it might be a major issue. For example, global-scale analyses have identified correlates of elevated extinction risk such as distribution size, insularity, body size, and ecology (Böhm et al. 2016; Ripple et al. 2017). Some studies have used these correlates to estimate proportions of data deficient species that are likely to be threatened (Tingley et al. 2013; Bland and Böhm 2016). Other expert panel-based analyses have also attempted to incorporate undescribed taxa into conservation prioritization exercises (Lintermans et al. 2020), thereby highlighting significant proportions of unnamed but threatened taxa. Across all analyses, small, and especially insular, distributions consistently emerge as key predictors of conservation concern. Indeed, insular areas such as the Pacific islands, Madagascar, and New Zealand have some of the most threatened biotas in the world (Carlquist 1974; Cheke and Hume 2008; Chapple et al. 2021). Accordingly, for taxa wherein insular distributions intersect with high levels of undescribed diversity and cryptic ecologies, CER may be acute.

**Fig. 1:**
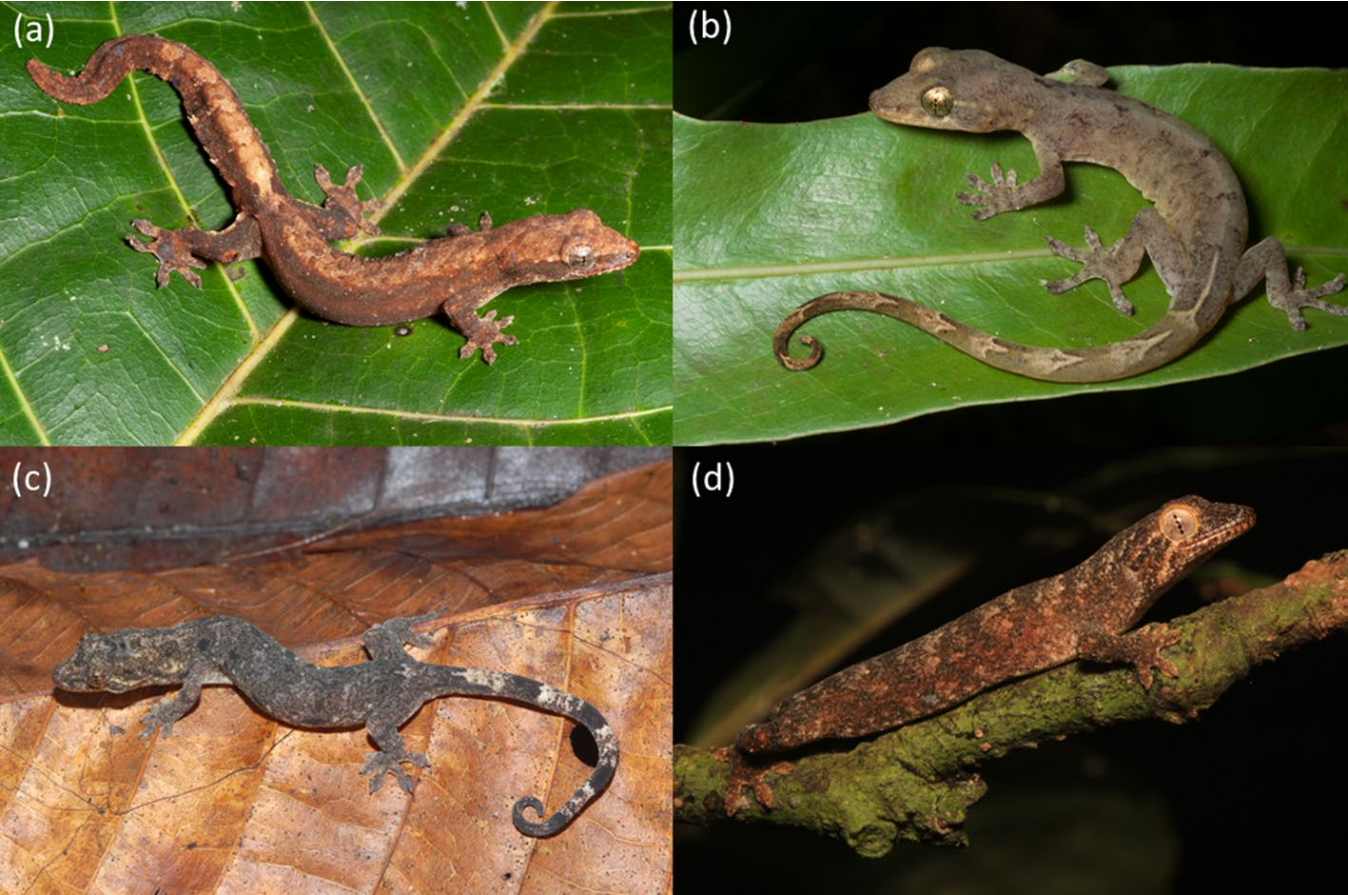
Examples of *Lepidodactylus*: **a** the newly described *Lepidodactylus bisakol* from the southern Bicol Peninsula, Philippines (Jason Fernandez and Rafe Brown); **b** the newly described *Pseudogekko hungkag* from Luzon Island, Philippines (Jason Fernandez and Rafe Brown); **c** an undescribed *Lepidodactylus* species from the Bismark Islands, Papua New Guinea (Steven Richards); and **d** *Lepidodactylus flaviocularis* known from only two specimens at one location on Guadalcanal, Solomon Islands (Scott Travers)

**Fig. 2:**
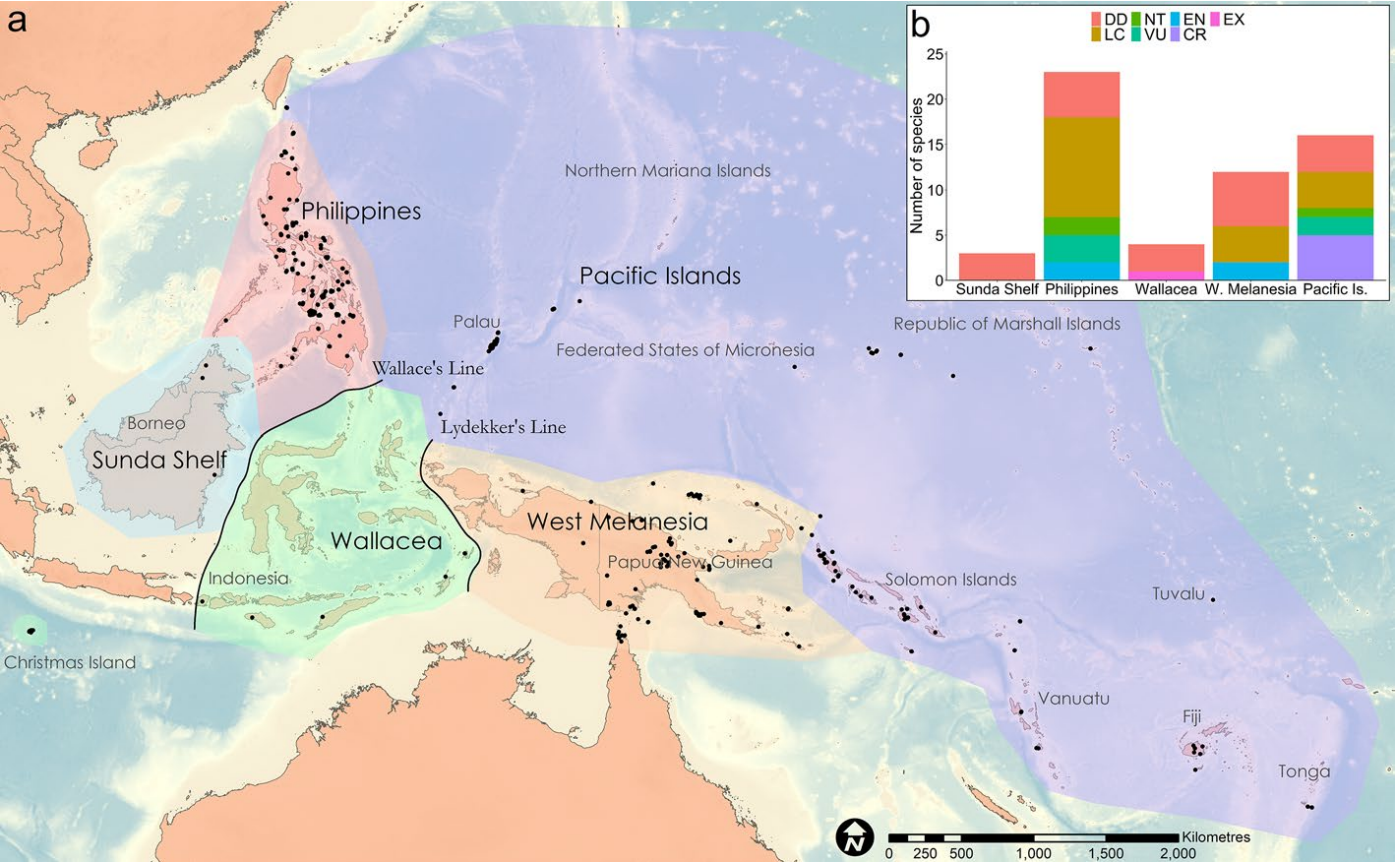
**a** Distribution of described *Lepidodactylus* species (black dots represent location records; www.gbif.org) across regions of the tropical western Pacific (excluding the widespread anthropogenically dispersed species *L. lugubris*). **b** Status of described *Lepidodactylus* species (n = 58) by region (*DD* data deficient, *LC* least concern, *NT* near threatened, *VU* vulnerable, *EN* endangered, *CR* critically endangered, *EX* Extinct)

The genus *Lepidodactylus* is a radiation of small (generally less the 10 cm adult body length), arboreal geckos with distributions spanning the tropical western Pacific (Figs. 1, 2; Oliver et al. 2018a). Recent phylogenetic analyses indicate that species currently placed in the genus *Lepidodactylus* are paraphyletic with respect to two lineages in the subgenus *Luperosaurus*, plus the entire genus *Pseudogekko* (Oliver et al. 2018a; Wood et al. 2020). Hereafter we use *Lepidodactylus* as a catchall for the combined clade of these lineages, comprising 58 described species currently, with phylogenetic analyses supporting a large number of additional, undescribed taxa (Oliver et al. 2018a; Eliades et al. 2021). In recent years, a suite of poorly known and/or highly restricted new *Lepidodactylus* species have also been described (Kraus 2019; Brown et al. 2020; Karkkainen et al. 2020; Eliades et al. 2021). A considerable, but as yet undocumented, number of described and candidate *Lepidodactylus* species are known only from small islands and atolls (e.g., Zug et al. 2003; Stubbs et al. 2017; Karin et al. 2018). Prior work on the *Lepidodactylus* also suggests that, like some other diverse insular lineages (Fernández‐Palacios et al. 2021; Richmond et al. 2021), this clade shows evidence of evolutionary displacement, with taxa concentrated away from species-rich lowland rainforests and into ‘marginal’ open, coastal or montane habitats, especially on the fringes of continental areas and larger islands like New Guinea and Borneo (Oliver et al. 2018a, 2020).

In addition to a pattern of evolutionary displacement around continental fringes, *Lepidodactylus* are secretive and for most species are rarely observed in the wild. As such, they are often the most poorly known reptile species where they occur (Brown and Acalala 1978; McCoy 2006). For example, there may be decades between species sightings despite substantial search effort in suitable habitat (Wiles and Conry 1990; Crombie and Menz 2007; Meneses et al. 2020), or observations may be infrequent especially at localities where other sympatric geckos are abundant (Bucol et al. 2012). Further highlighting their apparent rarity, several recent *Lepidodactylus* species descriptions are based on only one or two specimens collected from a single locality (Siler et al. 2014; Kraus 2019; Karkkainen et al. 2020; Eliades et al. 2021). *Lepidodactylus* species are also known from hard-to-access microhabitats, exemplified by the use of small hollow chambers within epiphytic ant plants and arboreal termitaria (Brown and Alcalala 1978; Ineich 2008, 2019; Oliver et al. 2015; Brown et al. 2020). The combination of distributional patterns (e.g., small islands, fringe habitats, and mountains), rarity, and microhabitat use, suggests an inherent vulnerability of *Lepidodactylus* species to major habitat disturbance events and ecological displacement from invasive species on contemporary ecological scales. Highlighting this vulnerability was the recent extinction from the wild of the Christmas Island endemic *Lepidodactylus listeri*, linked to predation by the introduced snake *Lycodon capucinus* and other invasive species (Emery et al. 2021). Christmas Island is an Indian Ocean Australian territory with well-resourced conservation management and locally based biologists. In contrast, most other *Lepidodactylus* occur in rarely monitored areas, suggesting that there is a high likelihood that the threat to this clade is overlooked or ‘cryptic’ and that other declines and extinction events may be occurring presently but, to date, remain undocumented.

Here we use *Lepidodactylus* as a model group for assessing approaches to estimate and highlight CER, herein broadly defined as underestimated or overlooked extinction risk within specific taxonomic groups and/or particular regions. We assembled an expert panel to: (a) update our knowledge of distributions, threats and status, (b) estimate the number of undescribed species, and (c) arrive at a final characterization of CER for species of *Lepidodactylus*. In the context of evolutionary and ecological displacement (Oliver et al. 2018a), we predicted that, compared to most other lizard groups, a greater proportion of *Lepidodactylus* species would be: (1) Data Deficient, (2) highly threatened by invasive species, (3) ecologically rare, (4) range restricted, and (5) ultimately, threatened with extinction.

## Methods

### IUCN red list assessment

We included all recognized species of *Lepidodactylus* and *Pseudogekko* in our assessment. For *Luperosaurus* species, we excluded taxa (*L. gulat, L. browni*, and *L. iskandari*) recently shown to be part of a separate radiation: the genus *Gekko* (Wood et al. 2020). However, we conservatively included all species of true *Luperosaurus* (members of the clade containing the type species of the genus: *L. cumingii*), even if their phylogenetic relationships remain untested. There were 58 recognized species in our focal group at the time we started this assessment (December 2020), of which 36 species had previously been assessed under IUCN Red List criteria (15 Least Concern (LC), 14 Data Deficient (DD), three Vulnerable (VU), two Endangered (EN), one Critically Endangered (CR), and one Extinct in the wild (EX)). Most had not been assessed since 2011 or earlier. Twelve of the recognized species have been described in the last decade, and none of these had been assessed.

We conducted a two-day virtual workshop to assess the extinction risk of all 58 described *Lepidodactylus* species against IUCN Red List criteria. The workshop was attended by facilitators from IUCN and Secretariat of the Pacific Regional Environment Programme (SPREP) and a panel of 11 experts (RB, FK, UA, SE, RF, MG, BK, CG, MS, CS, PO) with extensive knowledge of, and field experience with, the species assessed. Input from three non-attending experts (LG, II, SR) was subsequently incorporated as well. Prior to the workshop, the IUCN facilitator (PB) collated data reported for each species from all available literature (e.g., geographic range, population abundance, ecology, threats, and conservation measures), and entered these into the IUCN’s Species Information Service (SIS) database. Data were reviewed systematically (species-by-species) by the workshop panel, and augmented with input from all panellists, to arrive at final consensus, resulting in revised conservation status.

To broadly assess the degree to which *Lepidodactylus* may be less known and/or more threatened than other reptile taxa, we compared the percentages of threatened and data deficient species to similar studies, which have synthetically revised the conservation status of groups of reptiles (Böhm et al. 2013; Tingley et al. 2019; Chapple et al. 2021).

### Cryptic extinction risk

We compiled both published and unpublished genetic and morphological data to estimate the number of undescribed species of *Lepidodactylus*. Candidate species were included if they met one or more of the following criteria: (a) mitochondrial data indicating divergences (uncorrected p-distances) ≥ 10% (Oliver et al. 2009); (b) examination of relevant specimens, resulting in the combination of unique and diagnostic character sets (traditional categorical character state differences; or, in the case of measurements or meristic data, non-overlapping ranges of character values); or (c) unambiguous geographic disjunction from allied taxa coupled with photographic evidence of morphological distinctiveness. Descriptions of a number of these candidate species were in preparation at the time of our assessment (December 2020), and those taxa were still considered candidate species for this paper. We also emphasize that this list may be conservative. For example, there were no genetic or morphological data available for the widespread Fijian species *Lepidodactylus manni*; hence, this was considered as only one taxon (when we suspect it might constitute several species). Further, given the extreme rarity of many species (often known from a single specimen or locality), it seems likely that additional taxa remain completely overlooked. Conversely, we also emphasize that, for many of these candidate taxa, additional data will be required to validate the hypotheses that they represent distinct species. We are also aware of some recognised taxa that show very limited morphological or genetic divergence from other taxa, and may be synonyms (e.g., *Lepidodactylus browni* and *Lepidodactylus orientalis* or *Lepidodactylus pantai* and *Lepidodactylus woodfordi*; PMO pers. obs., Karin et al. 2021).

In addition to the data collected as part of our workshop assessment, we compiled a table of conservation-related attributes for all described and candidate species within *Lepidodactylus*, including: region (Sunda Shelf, including Borneo and Peninsular Malaysia; Philippines; Wallacea (based on phylogenetic evidence Christmas Island was considered part of Wallacea; Oliver et al. 2018a); West Melanesia, including Papua New Guinea (excluding Bougainville) and the Indonesian provinces of Papua and West Papua; and Pacific Islands, including Micronesia, Solomon Islands (including Bougainville), Vanuatu, Fiji, and Polynesia), extent of occurrence (area of minimum convex polygon around all location records or total land area where known only from an island < 1000 km^2^), number of specimens and sightings (from the GBIF and VertNet online databases and unpublished records from the panel), number of locations (minimum distance of 10 km separating locations, cf. Meiri et al. 2018), whether the taxon is restricted to small islands < 1000 km^2^, and year of last record. Consistent with Oliver et al. (2018a), we also recorded whether each species was known only from forest habitats (lowland rainforest, coastal forest, montane forest) versus also or only occurring in open habitats (e.g. beaches, disturbed anthropogenic landscapes or savannas), and whether the species is only known from lowland rainforest, a habitat type from which these geckos may be ecologically excluded in some areas (Oliver et al. 2018a, b).

### Data analysis

We used binary logistic regression models to assess the drivers of DD and threatened status in described *Lepidodactylus*. For the DD models, we assigned species as DD (1), or data sufficient (0) if LC, Near Threatened (NT), VU, EN, or EX. For the threatened species models, we assigned species as threatened (1) if VU, EN, CR or EX, or non-threatened (0) if NT or LC, and removed DD species. We selected covariates that were available for all taxa (including DD and candidate species), including region (categorical), number of locations (continuous; defined above), small-island distributions (categorical), year of last record (continuous), obligate forest dweller (categorical; defined above), and obligate lowland rainforest dweller (categorical; defined above). We were unable to include additional potentially useful covariates, such as phylogeny, as these data were unavailable for all taxa. We fitted individual covariate models in R (R Core Team 2020) and retained covariates that significantly predicted threatened status (p =  < 0.05). To avoid overfitting in the smaller data sufficient species pool, regions were fitted as individual covariates (e.g., Pacific Island region (1) versus other (0)). We modelled the retained covariates singularly and in all possible pairwise combinations, selecting the best-supported model as the highest R^2^ with a difference in Akaike information criterion (AIC) from the best-ranked model of < 2.0 (Symonds and Moussalli 2011).

To predict the numbers of DD and candidate species that are threatened, we applied the best-supported threatened logistic regression model using the predict () function in R, assigning species with a probability > 0.5 as potentially threatened.

## Results

### IUCN red list assessment

We found twenty-one (36%) of the described *Lepidodactylus* species to be DD, with insufficient data preventing adequate assessment of conservation status. This percentage is higher than the global percentages of DD reptiles reported at the Order level (0–24%, except for Amphisbaenia) and realm (0–33%) (Böhm et al. 2013), higher than any of the world’s skink subfamilies (3–15%) (Chapple et al. 2021), higher than any reptile family in Australia (0–18%) (Tingley et al. 2019), and higher than 25 of 26 reptile Families in Tanzania (0–27%). West Melanesia had the highest number of DD species (n = 6) (Fig. 2). Region was the best-supported logistic regression model predicting DD status (Table 1), with DD species more likely to occur on Sunda Shelf than the Philippines or Pacific Islands (Fig. 2).

**Table 1:** Binary logistic regression models predicting a Data Deficient versus threatened status (VU, EN, CR) in described species of the *Lepidodactylus*

Model	AIC	dAIC	R^2^
Data deficient			
DD ~ Region	78.37	0	20.4
Threatened			
Threatened ~ Locations	49.24	0	21.9
Threatened ~ Locations + Small_island	49.38	0.14	25.7
Threatened ~ Small_island	53.07	3.83	13.3

We found fifteen (41%, calculated as (VU + EN + CR + EX)/(Total N-DD)) described *Lepidodactylus* species were threatened (VU, EN, CR) or extinct (EX). This percentage is higher than the global percentage of threatened reptiles reported for all reptile orders (7–21%) and all biogeographic realms (12–25%) except Oceania (42.9%) (Böhm et al. 2013), higher than any of the world’s skink subfamilies (0–30%) (Chapple et al. 2021), and higher than any reptile family in Australia (0–17%) (Tingley et al. 2019). The Pacific Islands region had the most threatened species, including five Critically Endangered, and two Vulnerable species (Fig. 2). The Philippines had the next highest number of threatened species, with two endemic taxa categorized as Endangered and three assessed as Vulnerable (Fig. 2). Number of locations and small-island endemism were included in the best-support logistic regression model to predict threat status (Table 1). Threatened species were recorded from significantly fewer locations than non-threatened species and were more likely to occur on small islands < 1000 km^2^ in area. There has been one documented *Lepidodactylus* extinction, with the Christmas Island endemic *Lepidodactylus listeri* last recorded in the wild in 2012.

Of the 58 described *Lepidodactylus* species, 33% (n = 19) are known only from their type localities. This is a higher percentage than previously documented in each of the world’s six gecko families (8–23%) and higher than in all but two of the world’s 42 lizard families (Meiri et al. 2018). Further, fewer than 50% of *Lepidodactylus* species are known from more than two locations. There was no significant difference in average extent of occurrence (EOO) across our five regions (Kruskal–Wallis chi squared = 3.71, df = 3, p = 0.30) for the 53 species with these data available. Ten (17%) of the described *Lepidodactylus* species have not been recorded in the last 20 years, including (date of last record shown in parentheses for each species): *L. dialeukos* (1938), *L. euaensis* (1992), *L. gardineri* (1982), *L. labialis* (1971), *L. mutahi* (1966), *L. oortii* (1923), *L. shebae* (1944), *L. tepukapili* (1998), *L. zweifeli* (1969), and *Luperosaurus yasumai* (1994).

**Fig. 3:**
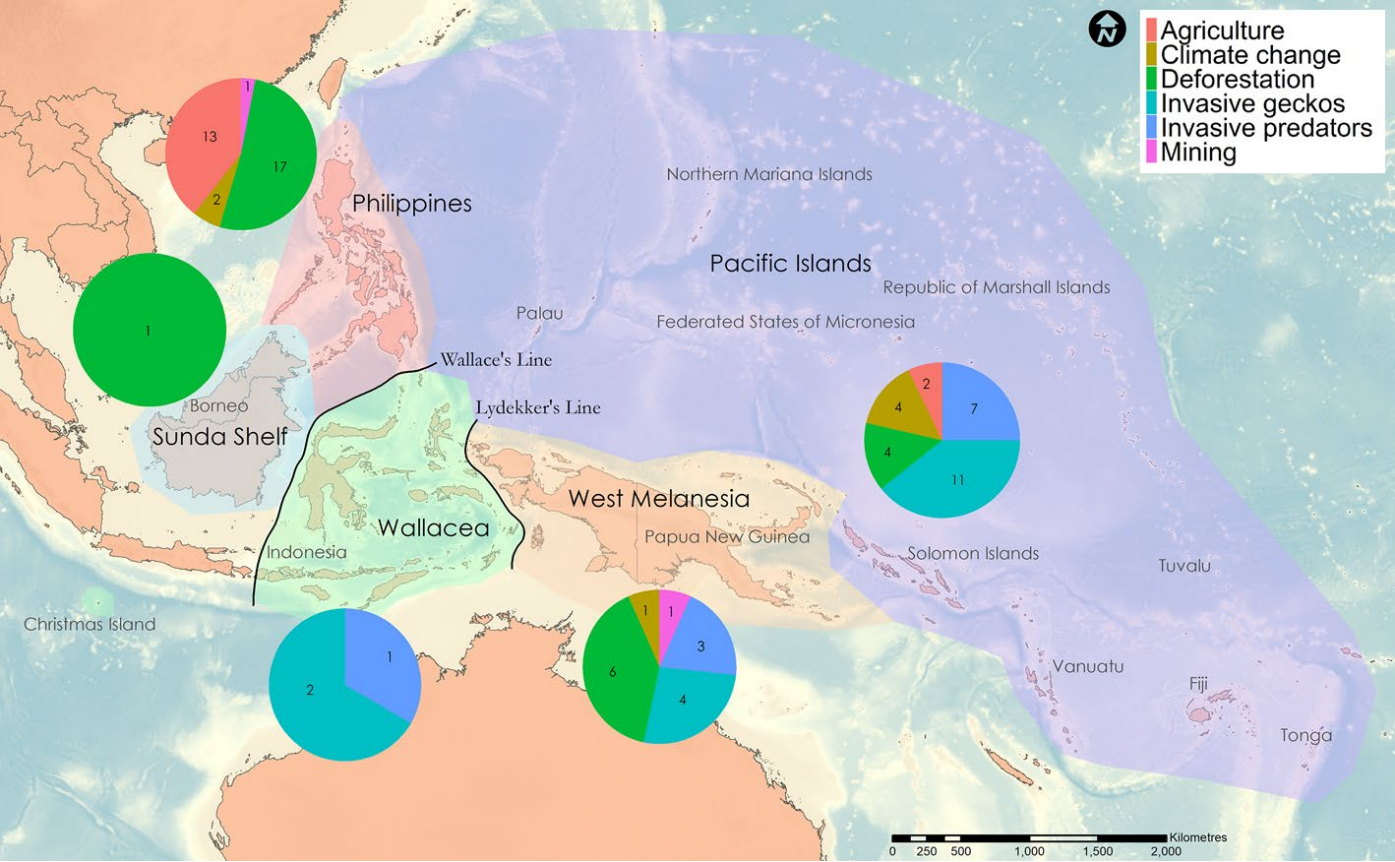
Distribution of key threats nominated to described *Lepidodactylus* species across regions of the tropical western Pacific, including numbers of species affected by each threat. Note that not all species have listed threats and some species have multiple threats, thus total numbers across threat categories will not match the number of species in each region

The main listed threats to *Lepidodactylus* are not evenly distributed across the regions (Fig. 3). Specifically, deforestation, and agriculture were the dominant threats in the two western regions (Sunda Shelf and the Philippines) and in West Melanesia, whereas invasive geckos and predators were dominant threats east of Wallace’s Line (Fig. 3).

### Cryptic extinction risk

**Fig. 4:**
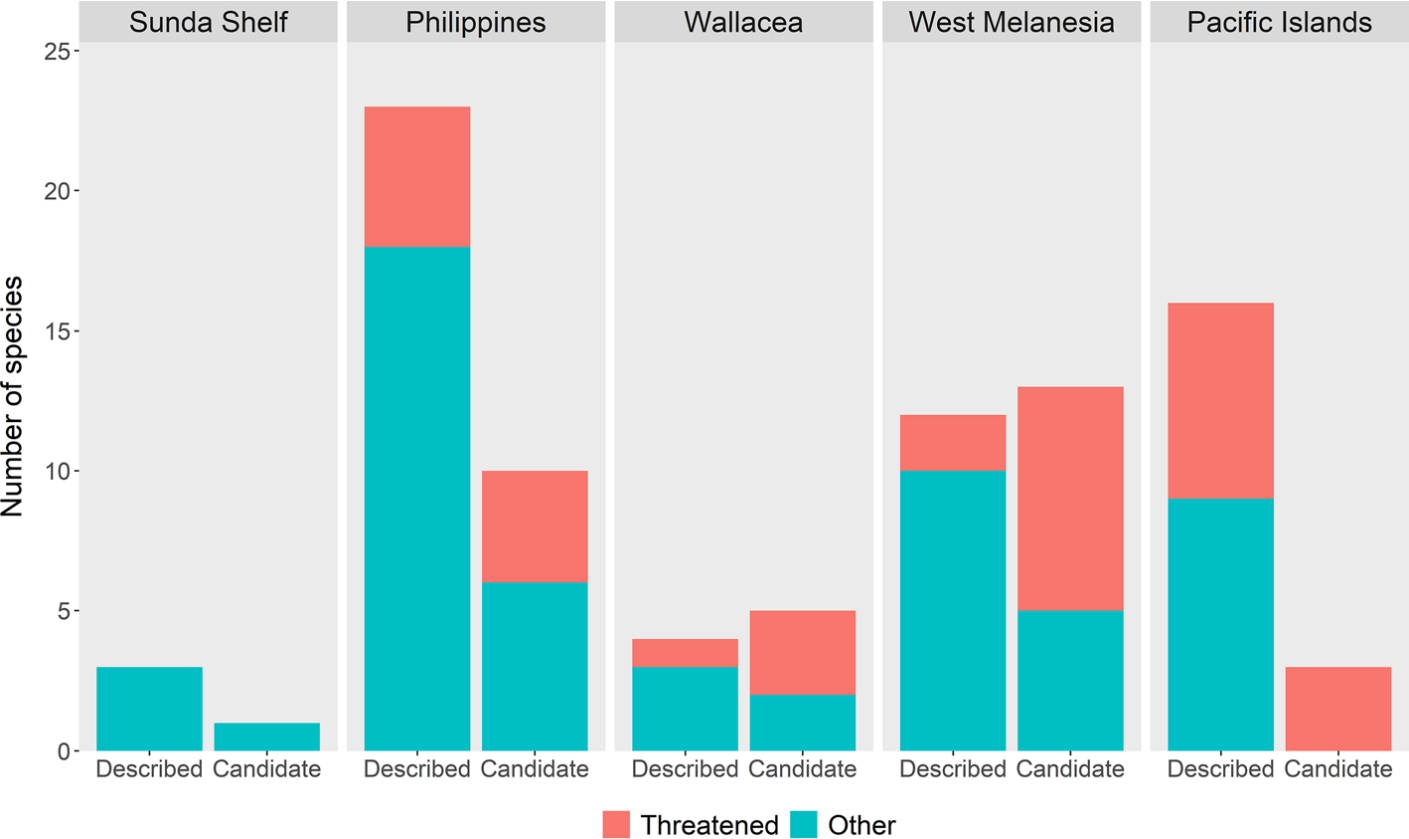
Numbers of described and candidate *Lepidodactylus* species by region (indicated above each column) and threat status (colours; see key below) in the tropical western Pacific. ‘Threatened’ (VU, EN, CR) and ‘Other’ (DD, LC, NT) candidate species were assigned based on our logistic regression model

Our panel identified 32 candidate *Lepidodactylus* species in addition to the 58 currently described, which is 35% of known species diversity in this clade. Candidate species were non-randomly distributed, with the highest numbers in West Melanesia (n = 13) and the Philippines (n = 10) (Fig. 4). West Melanesia and Wallacea each had more candidate species than described species (Fig. 4). The Pacific Islands had the lowest number of candidates relative to described species (3 versus 16) (Fig. 4.). Twenty of these 32 candidate species have been included in a published clade-wide phylogeny (Oliver et al. 2018a).

Half (n = 16) of these candidate species were only known from islands of < 1000 km^2^, and 78% (n = 25) are known from only one location. Candidate species were also typically rare despite substantial search effort involving many person hours or search nights per specimen (e.g. Eliades et al. 2021). Most of the candidate species have been collected on relatively recent expeditions, with 84% (n = 27) first collected post-2000 and 63% (n = 20) collected post-2010 (Table S1), and 78% (n = 25) of candidate species have already been referred to in the published literature.

Our threatened status logistic regression model predicted an additional three and 15 threatened species from the DD and candidate species pools, respectively (Table 2). Eight of the taxa predicted to be threatened are from West Melanesia, four are from the Philippines, and three are from each of Wallacea and the Pacific Islands (Table S1). Priority *Lepidodactylus* species for conservation and field surveys comprise 36.7% of all species in the clade (Tables 2, S1).

**Table 2:** Numbers of *Lepidodactylus* species assessed or predicted as threatened (Vulnerable, Endangered, Critically Endangered, or Extinct)

Taxa	Total number of species	Number of species listed or predicted threatened	% Species threatened
Data sufficient species prior to this study	22	6	27.3
Data sufficient species (all)	37	15	40.5
Data deficient species	21	3	14.3
Candidate species	32	15	46.9
Total	90	33	36.7

## Discussion

*Lepidodactylus* geckos are relatively highly threatened, both in terms of the number and percentage of taxa of concern; importantly, the number of threatened species was underestimated prior to this study. Our results demonstrate the value of combining standard, predominately range-based IUCN Red List assessments of described taxa with explicit consideration of what we term cryptic extinction risk. Specifically, although our updated conservation assessment of recognised species more than doubled the number of threatened species (from six to 15), this estimate was again more than doubled (to 33) through our model predictions of threatened status in Data Deficient and undescribed candidate species. Considering CER in conservation assessment could benefit other inherently vulnerable biotic groups with high levels of documented, but undescribed, taxonomic diversity.

### Cryptic extinction risk

Our work provides a demonstration of the extent to which the Linnean Shortfall and other knowledge gaps (e.g. Wallacean Shortfall) have led to underestimation of extinction risk in an exemplary clade of poorly known species, typified by cryptic ecology and underestimated species diversity. Although *Lepidodactylus* species are relatively small lizards and often an inconspicuous part of many lizard communities across our study region (Brown et al. 2013; Sanguila et al. 2016), our data indicate that, with close to one hundred species, *Lepidodactylus* is the most diverse gecko radiation across the Philippines and much of the Pacific. Our panel also noted that many *Lepidodactylus* are exceedingly rare (e.g., *Lepidodactylus flaviocularis*, *Luperosaurus* spp. on large islands; Oliver et al. 2020; Eliades et al. 2021), especially on larger islands where there are richer gecko assemblages and on small islands, in the presence of invasive geckos (e.g., *Hemidactylus* spp.). This scarcity likely reflects genuine rarity or species’ reliance on difficult-to-access microhabitats (Grismer 2011; Oliver et al. 2015; Brown et al. 2020; Eliades et al. 2021). Either possibility could also be linked to ecological displacement and/or susceptibility to predation. A tendency for *Lepidodactylus* species to be ecologically displaced is consistent with broader and deeper patterns of evolutionary displacement around continental fringes (Oliver et al. 2018a, b, 2020). We argue that this combination of cryptic ecology, ecological vulnerability, and high levels of unrecognised species diversity has led to a serious and likely ongoing underestimation of both the number and percentage of *Lepidodactylus* species that are threatened—and potentially already extinct (see below).

Our approach to estimating threatened status differs from other assessment methods of particular biotic groups, such as expert elicitation of extinction percentage risk (Lintermans et al. 2020; Geyle et al. 2021), in that potential at-risk taxa can be identified from single species’ geographical occurences and few other data. Our approach is also distinct from other methods seeking to overcome the taxonomic impediment, such as mapping centers of phylogenetic endemism (Rosauer et al. 2018), because data deficient and undescribed candidate species from small islands can be identified as priorities for conservation management without any phylogenetic data, or overlap with significant hotspots of diversity or distinctiveness. Consequently, our method of assessing CER may be most appropriate for insular species groups that combine traits such as documented but undescribed diversity, many localized endemics, and often highly cryptic ecologies. An obvious further candidate group would be the other major Pacific lizard radiation of skinks in the genus *Emoia*. It may be also applicable to some invertebrate groups such as mygalomorph spiders wherein described diversity has been documented and many taxa show localised ranges (e.g. Rix et al. 2020).

### Spatial patterns of extinction risk and threats

We found that extinction risk in *Lepidodactylus* was not randomly distributed but concentrated in small-island systems, especially the Pacific Islands region and, to a lesser extent, West Melanesia and Wallacea. This geographic variation in vulnerability is consistent with research on Pacific island birds demonstrating frequent losses of more than 50% of bird species on islands throughout the western Pacific following the arrival of humans (Steadman 2006). Some islands of Wallacea also show evidence of Holocene extinction and turnover in key faunal elements (Turvey et al. 2017; Louys et al. 2021). Although fossil remains of smaller lizards are uncommon on these islands, prehistoric extinctions of unassignable gecko taxa have been documented in Fiji, Tonga and the Mariana Islands (Pregill 1993, 1998; Pregill and Steadman 2014). More recently, extirpations of several small gecko species have been recorded throughout the tropical northwest Pacific (Pregill and Steadman 2009). These observations, together with the recent extinction of *Lepidodactylus listeri* from Christmas Island (Andrew et al. 2018), the highly isolated and localized distributions of many taxa (e.g., *Lepidodactylus paurolepis* from Palau and *L. oligoporus* from the Mortlock Islands), and the number of undescribed species from poorly sampled locations (Eliades et al. 2021), point to the loss of a much richer evolutionary history than has been documented (Oliver et al. 2018a), and a likelihood of future extinction events in these regions. Even during this work we gathered reports that the Rotuma Island endemic (*Lepidodactylus gardneri*) has not been recently seen despite active searching (Monifa Fiu, pers. obs.), raising grave concerns about the persistence of this small island endemic.

As with extinction risk, we also found that threats to *Lepidodactylus* species were not randomly distributed. A dominant role of invasive species was identified east of Wallace’s Line and especially in the Pacific Islands, where many small-island endemic *Lepidodactylus* species occur. The importance of invasive species is consistent with the idea that *Lepidodactylus* are particularly susceptible to novel predators and ecological displacement, and supported by the likely role of an introduced snake (*Lycodon capucinus*) in the recent extinction of *Lepidodactylus listeri* (Emery et al. 2021). In this context, the human-assisted expansion of *L. capucinus* east of Wallace’s Line, through the Lesser Sundas and into islands around New Guinea (O’Shea et al. 2018) is particularly concerning. In contrast, in more western regions and on larger landmasses, habitat loss through deforestation was the major threat to the many obligate forest-dwelling *Lepidodactylus* species (e.g., Das et al. 2008; Siler et al. 2014; Kraus and Oliver 2019; Brown et al. 2020; Eliades et al. 2021), a pattern consistent with global trends for small vertebrates (Ripple et al. 2017).

### Steps towards understanding and conserving biodiversity in rare and undescribed taxa

The true conservation status of a significant majority (59%) of *Lepidodactylus* taxa (candidate and Data Deficient species) remains unknown. Many species are very difficult to find, let alone effectively monitor (Brown and Alcala 1978). Thus, although we encourage targeted searches, novel sampling methods (e.g., Bell 2009), and efforts to identify key microhabitats (Brown et al. 2013; Sanguila et al. 2016), reward per unit effort is likely to be low, especially for rare species from larger island systems (e.g., Sunda Shelf and the Philippines) (Eliades et al. 2021). Many key areas that need to be surveyed are also both logistically and politically challenging to access (e.g., *Luperosaurus joloensis*, known only from four specimens, collected from three locality es in the Sulu Archipelago and Western Mindanao Island; Supsup et al. 2020). Given these limitations, the following research and conservation actions may further knowledge of *Lepidodactylus* diversity and enhance species conservation:

1. *Integrative and innovative approaches to resolving species diversity.* This is particularly important for an array of remote island taxa from the Pacific. Relatively new techniques for systematics such as target capture genomic studies for obtaining DNA from old specimens with degraded DNA and CT scanning may provide avenues to resolve species status for taxa known from few or very old specimens. Although it is preferable to describe species based on a robust, statistical sample size and vouchered series of genotyped animals, the extreme rarity of many *Lepidodactylus* species suggests this will often not be possible, and taxonomic studies should proceed, providing there is no doubt of evolutionary distinctiveness. A complete phylogentic picture for the clade would also allow for phylogeny to be included in models predicting threatened or DD status, perhaps improving the accuracy of predictions.
2. *Targeted surveys*. Although *Lepidodactylus* species are extremely difficult to detect in many ecological systems, targeteted surveys on remote islands or in habitats that may serve as refuges for vulnerable species could be informative (Table S1). Numerous small-island endemics from the Pacific urgently require re-survey to confirm that they have not been extirpated (e.g., *Lepidodactylus gardineri*, *L. oligoporus*, *L. tepukapili*, and *L.* sp. Nuguria). We again note that recent biodiversity surveys have failed to locate *L. gardineri* in Rotuma (Monifa Fiu, *pers. comm.*). Several species, known only from small islands, are also surprisingly abundant on these islands (e.g., *Lepidodactylus mitchelli* on Boia Boia Waga Island, *L.* sp. Kur on Kur Island, and *Luperosaurus macgregori* on Babuyan Claro Island). These specific cases may provide instances for which more meaningful data on population trends could illuminate key ecological interactions associated with long-term persistence of unique species on isolated small islands.
3. *Building within-region expertise to monitor species*. The (apparent) last refuges of many *Lepidodactylus* taxa are remote and difficult to access. This situation has only been compounded by the Covid-19 pandemic, with restrictions making travel difficult or impossible throughout the region. Building and supporting local expertise and capacity may effectively address these issues and could also strengthen the ownership countries have in protecting their threatened endemic species.
4. *Strengthen quarantine safeguards on remaining island refuges*. Invasive species are almost certainly the most important threat to many small-island *Lepidodactylus* species. On islands that are frequently visited by people effective quarantine is challenging. For remote islands (e.g., Rotuma, Tuvalu and Tonium Island), however, strategies to prevent colonization by invasive rats, wolf snakes (*Lycodon*), common house geckos, and non-native lizards may be the only feasible strategy for preventing the extinction of many endemic species. Such strategies are currently being funded and implemented in the Pacific Islands region (e.g., PRISMSS Programme www.sprep.org/prismss/protect-our-islands), though awareness of protecting small-island endemics, such as *Lepidodactylus*, needs to be raised.
5. *Habitat protection.* Indonesia and Malaysia are among the top ten countries for global forest loss since 2000, and rates of deforestation have accelerated over the last decade in Papua New Guinea and the Solomon Islands (Global Forest Watch 2014). Highlighting the threats to forest-dwelling *Lepidodactylus* in Melanesia is the case of Woodlark Island (874 km^2^) in PNG, inhabited by *L. kwasnickae* and at least 47 other endemic plant and animal species, wherein there are plans for near complete deforestation for palm oil plantations (Kraus 2021). Expansion of the terrestrial protected area in these countries, all of which fell short of the Aichi Target of 17% of terrestrial area by 2020 (Malaysia 13.3%, Indonesia 12.2%, PNG 3.7%, Solomon Islands 1.8%; UNEP-WCMC and IUCN 2020), could help to secure the futures of forest-dwelling *Lepidodactylus*, and other endemic species, particularly in lowland rainforests and coastal habitats.

## Conclusion

Our data on *Lepidodactylus* highlight how a combination of cryptic ecologies and unrecognized species diversity can lead to significant underestimation of conservation threat, here termed Cryptic Extinction Risk (CER). Accounting for CER in conservation assessment thus has the potential to highlight at-risk taxa and geographic areas of the concern, which may otherwise be overlooked. Our results also highlight the urgency for surveys targeting threatened Pacific Island *Lepidodactylus* species, as well as the importance of strengthening quarantine safeguards in this region. The CER may be useful for highlighting conservation issues in other taxa that combine aspects of a workable but incomplete taxonomic framework, poorly documented geographical distributions, many localized endemics, and cryptic or otherwise poorly known ecologies.

## Supplementary Information

Below is the link to the electronic supplementary material.

Supplementary file1 (DOCX 22 kb)

## Data Availability

Data are available in the supplementary material, IUCN Red List, Global Biodiversity Information Facility and on request to authors.
