# Supplementary material for: Cryptic extinction risk in a western Pacific lizard radiation
Source: Biodivers Conserv. 2022 May 25;31(8-9):2045–62. doi: 10.1007/s10531-022-02412-x (PMC9130968; doi:10.1007/s10531-022-02412-x)
Supplement: Supplementary file 1 — Supplementary file1 (DOCX 22 kb) [file 10531_2022_2412_MOESM1_ESM.docx]

Table S1. Priority conservation *Lepidodactylus* clade species

|  | | |  | |  | |  |  |  |
| --- | --- | --- | --- | --- | --- | --- | --- | --- | --- |
| **Species** | **Red List status** | **Region** | | **Last recorded** | | **Restricted to small islands (<1000 km2)** | **Restricted to forest** | **Restricted to tropical lowland forest** |  |
| **Threatened species** |  |  | |  | |  |  |  |  |
| *Lepidodactylus flaviocularis* | CR | Pacific Islands | | 2014 | | No | 1 | 1 |  |
| *Lepidodactylus gardineri* | CR | Pacific Islands | | 1982 | | Yes | 1 | 0 |  |
| *Lepidodactylus kwasnickae* | EN | West Melanesia | | 2011 | | Yes | 1 | 1 |  |
| *Lepidodactylus listeri* | EX | Wallacea | | 2012 | | Yes | 1 | 0 |  |
| *Lepidodactylus oligoporus* | CR | Pacific Islands | | 2004 | | Yes | 0 | 0 |  |
| *Lepidodactylus paurolepis* | VU | Pacific Islands | | 2004 | | Yes | 0 | 0 |  |
| *Lepidodactylus manni* | VU | Pacific Islands | | 2018 | | No | 1 | 1 |  |
| *Lepidodactylus euaensis* | CR | Pacific Islands | | 1992 | | Yes | 1 | 0 |  |
| *Lepidodactylus tepukapili* | CR | Pacific Islands | | 1998 | | Yes | 0 | 0 |  |
| *Lepidodactylus zweifeli* | EN | West Melanesia | | 1969 | | No | 1 | 0 |  |
| *Luperosaurus joloensis* | EN | Philippines | | 2009 | | No | 1 | 0 |  |
| *Pseudogekko brevipes* | VU | Philippines | | 2002 | | No | 1 | 1 |  |
| *Pseudogekko hungkag* | VU | Philippines | | 2017 | | No | 1 | 1 |  |
| *Pseudogekko isapa* | VU | Philippines | | 2014 | | No | 1 | 1 |  |
| *Pseudogekko sumiklab* | EN | Philippines | | 2017 | | No | 1 | 0 |  |
| **Data deficient species predicted threatened** |  |  | |  | |  |  |  |  |
| *Lepidodactylus aignus* | DD | West Melanesia | | 2003 | | Yes | 1 | 0 |  |
| *Lepidodactylus mitchelli* | DD | West Melanesia | | 2015 | | Yes | 0 | 0 |  |
| *Lepidodactylus pollostos* | DD | West Melanesia | | 2005 | | Yes | 1 | 0 |  |
| **Candidate species predicted threatened** |  |  | |  | |  |  |  |  |
| *Pseudogekko* cf *compressicorpus* | NA | Philippines | | 2014 | | Yes | NR | NR |  |
| *Lepidodactylus* sp. Wallis and Futuna | NA | Pacific Islands | | 2013 | | Yes | NR | NR |  |
| *Lepidodactylus* sp. Banda | NA | Wallacea | | 2011 | | Yes | NR | NR |  |
| *Lepidodactylus* sp. Kei Kecil | NA | Wallacea | | 2014 | | Yes | NR | NR |  |
| *Lepidodactylus* sp. Kur | NA | Wallacea | | 2014 | | Yes | NR | NR |  |
| *Lepidodactylus* sp. Luzon | NA | Philippines | | 2018 | | Yes | NR | NR |  |
| *Lepidodactylus* sp. Tonga | NA | Pacific Islands | | 1968 | | Yes | NR | NR |  |
| *Lepidodactylus* sp. Ndrova | NA | West Melanesia | | 1973 | | Yes | NR | NR |  |
| *Lepidodactylus* sp. Nuguria | NA | West Melanesia | | 1978 | | Yes | NR | NR |  |
| *Lepidodactylus* sp. Philippines widespread | NA | Philippines | | 2018 | | Yes | NR | NR |  |
| *Lepidodactylus* sp. Rennell | NA | Pacific Islands | | 2018 | | Yes | NR | NR |  |
| *Lepidodactylus* sp. Sudest | NA | West Melanesia | | 2004 | | Yes | NR | NR |  |
| *Lepidodactylus* sp. Bismarck Sea 1 | NA | West Melanesia | | 2018 | | Yes | NR | NR |  |
| *Lepidodactylus* sp. Bismarck Sea 2 | NA | West Melanesia | | 2018 | | Yes | NR | NR |  |
| *Luperosaurus* sp. Sibuyan | NA | Philippines | | 2017 | | Yes | NR | NR |  |
| **Other species and candidates not recorded in >30 years** |  |  | |  | |  | NR | NR |  |
| *Lepidodactylus oorti* | DD | Wallacea | | 1923 | | No | 0 | 0 |  |
| *Lepidodactylus dialeukos* | DD | West Melanesia | | 1938 | | No | 1 | 1 |  |
| *Lepidodactylus mutahi* | DD | West Melanesia | | 1966 | | No | 1 | 1 |  |
| *Lepidodactylus labialis* | LC | Philippines | | 1971 | | No | 1 | 0 |  |
| *Lepidodactylus* sp. Waigeo | NA | West Melanesia | | 1974 | | No | NR | NR |  |
